# Sensitivity and specificity of tuberculosis signs and symptoms screening and adjunct role of social pathology characteristics in predicting bacteriologically confirmed tuberculosis in Myanmar

**DOI:** 10.1186/s41182-020-00292-x

**Published:** 2021-01-07

**Authors:** Kyaw Ko Ko Htet, Virasakdi Chongsuvivatwong, Si Thu Aung

**Affiliations:** 1 Department of Medical Research, Ministry of Health and Sports, Pyin Oo Lwin, Myanmar; 2 Epidemiology Unit, Faculty of Medicine, Prince of Songkla University, Hat Yai, 90110 Thailand; 3 Department of Public Health, Ministry of Health and Sports, Nay Pyi Taw, Myanmar

**Keywords:** TB signs and symptoms, Sensitivity and specificity, Social pathology, Screening

## Abstract

**Background:**

Globally, using tuberculosis signs and symptoms (TB-SS) as a screening tool has become less important due to its low sensitivity and specificity. We analyzed data from the Myanmar National Tuberculosis (TB) prevalence survey in 2010. The various TB screening models were developed to predict TB by using logistic regression analysis, and their performance on TB prediction was compared by the measures of overall performance, calibration and discrimination ability, and sensitivity and specificity to determine whether social pathology characteristics could be used as a TB screening tool.

**Results:**

Among 51,367 participants, 311 (0.6%) had bacteriologically confirmed TB, of which 37.2% were asymptomatic and 2% had a normal chest X-ray. Out of 32 various combinations of signs and symptoms, having any signs and symptoms gave the best sensitivity of 59.8% and specificity of 67.2%, but chest X-ray (CXR) alone gave the highest sensitivity (95.1%) and specificity (86.3%). The next best combination was cough only with a sensitivity of 24.4% and specificity of 85%. Other combinations had poor sensitivity (< 10%). Among various TB screening models, the overall performance *R*^2^ was higher in the combined models of social pathology and TB signs and symptoms as well as the social pathology model, compared to TB-SS models (> 10% versus < 3%), although all TB screening models were perfect to predict TB (Brier score = 0). The social pathology model shows a better calibration, more closer to 45° line of calibration plot with Hosmer-Lemeshow test *p* value = 0.787, than the combined models while it had a better discrimination ability in area under the curve, AUC = 80.4%, compared to TB-SS models with any signs and symptoms, AUC = 63.5% and with any cough, AUC = 57.1% (DeLong *p* value = 0.0001). Moreover, at the propensity score cutoff value ≥ 0.0053, the combined and social pathology models had sensitivity of ~ 80% and specificity of ~ 70%. The highest population attributable fraction to predict TB by social pathology characteristics was male gender (42.6%), age ≥ 55 years (31.0%), and underweight (30.4%).

**Conclusion:**

Over one-third of bacteriologically confirmed TB was asymptomatic. The conventional TB-SS screening tool using any TB signs and symptoms had a lower sensitivity and specificity compared to CXR and social pathology screening tools. The social pathology characteristics as TB screening tool had good calibration and can improve the discrimination ability to predict TB than TB-SS screenings and should be encouraged.

## Background

Early detection and initiation of treatment of all tuberculosis (TB) patients is necessary to reduce mortality, morbidity, and transmission in the community [1]. Screening for tuberculosis signs and symptoms (TB-SS), such as cough, hemoptysis, loss of weight, chest pain, fever, night sweat, and shortness of breath, was a key component of the National TB Strategy for combating TB. In the current global practice, signs and symptoms screening is the first step for TB case finding, and those who screen positive are recommended to have a chest X-ray (CXR) and sputum smear examination [2, 3]. However, a review on the National TB prevalence survey in Asia (1990–2012) revealed that 40–79% of TB cases were asymptomatic [4]. Therefore, using signs and symptoms as a screening tool is still a global challenge because it happens missing TB cases in the community [5].

Globally, the slow reduction in TB incidence has prompted a search for a new approach in TB intervention [6]. As the current approach, TB is regarded as a medical disease. Therefore, TB screening is relying on TB-SS. In new point of view, TB is considered as the social pathology disease because it is associated with people’s social, biological, and pathological characteristics [7–11]. Those characteristics included age, gender, occupation and economic status, smoking and alcohol, contact to index TB patients, human immunodeficiency virus, diabetes mellitus and malnutrition, crowding, and poor ventilation [12–21].

As people has risk of acquiring TB infection under relationship with social pathology characteristics, taking into account those characteristics in TB screening tool would be a potential new approach to improve TB case detection. However, performance in predicting TB case detection by using social pathology characteristics compared to TB-SS screening still needs to be evaluated. Therefore, our study was performed with the objectives of determining (i) the sensitivity and specificity of various combinations of TB-SS and (ii) the adjunctive role of social pathology characteristics in TB screening, compared with TB-SS for improving TB cases detection.

## Methods

### Study design

We revisited the data set of the National TB prevalence survey 2010, Myanmar. The analysis was done with the approval from the National Tuberculosis Programme.

### Study setting

#### General setting

Myanmar is one of the 30 high TB burden countries ranking 11th globally and 4th in the South-East Asia region after India, Bangladesh, and Indonesia. In 2016 in Myanmar, an estimated 191,000 people developed TB of whom 30,000 died [22].

#### Specific setting

##### Screening and diagnostic process of participants during the National TB prevalence survey

**Fig. 1:**
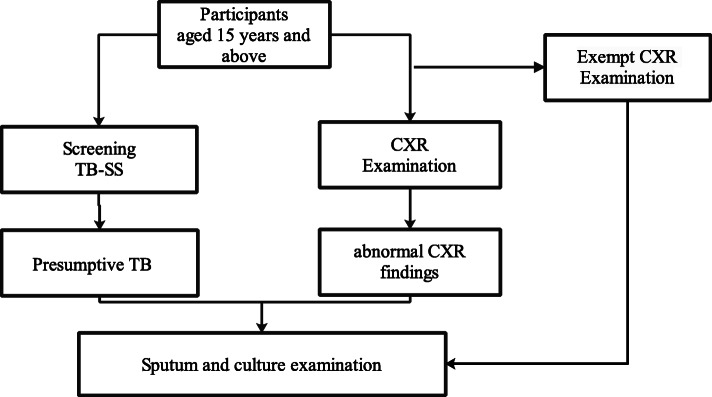
Survey process for screening and diagnosis of TB among eligible participants of the household during the National TB prevalence survey. TB, tuberculosis; TB-SS, tuberculosis signs and symptoms; CXR, chest X-ray

Figure 1 reveals the survey process for screening and diagnosis of TB among eligible household members during the National TB prevalence survey. Individual interviews emphasizing on TB-SS within the previous month and mobile CXR examinations were used as a parallel screening tool among survey participants for processing bacteriological examinations.

All interviewees except those with a first-trimester pregnancy were screened with CXR examinations. Pregnant women who had been excluded from the CXR examination underwent a compulsory sputum examination for smear and culture. Those suspected of having TB were screened with a CXR and if abnormal radiological findings were detected, underwent sputum examination of smear and culture.

The presumptive TB with signs and symptoms were sent for sputum examination of smear and culture after their CXR, regardless of the CXR results. The participants with both normal CXR finding and without TB-SS were categorized as normal healthy participants without continuing any confirmation test as the World Health Organization (WHO) guideline [23]. Those who did not appear at the survey site were revisited by the team, and transportation was arranged for CXR examination if necessary. Where possible, the team took sputum specimens from those who could not be screened by CXR.

### Data variables

The main outcome variable was bacteriologically confirmed TB which is used as the gold standard for calculating the sensitivity and specificity of various combination of TB signs and symptoms and used for predicting TB by adjusting covariates.

There were 18 independent predictor variables for TB detection: (i) social characteristics: age group (years), gender, education, occupation, religion, marital status, area of residence (rural or urban), administrative division (region or state), smoking and drinking, contact with a known TB case, previous history of TB but no current treatment; (ii) pathology characteristics: body mass index (kg/m^2^), diabetes mellitus, hypertension, and human immunodeficiency virus (HIV). The 14 administrative divisions of Myanmar were classified into two groups based on whether the majority of people living there were of Bamar ethnicity (region) or belonged to an ethnic minority group (state). Body mass index was categorized as underweight (< 18.5 kg/m^2^), normal (18.5–24.9 kg/m^2^), and overweight/obese (≥ 25 kg/m^2^) [24] and (iii) TB-SS included cough, hemoptysis, recent loss of weight, chest pain, and fever within previous 1 month. If one of the TB-SS was present, it was regarded as any TB signs and symptoms variable. If there was cough with any duration but does not have any other symptoms, it was defined as any cough variable.

### Data analysis and statistics

The data was analyzed in R studio using R version 4.0.0 (the R foundation for Statistical Computing) [25]. The prevalence of bacteriologically confirmed TB was summarized per 100,000 population based on TB signs and symptoms and chest X-ray as a parallel screening tool.

The situation of TB was tabulated against various combinations of TB-SS. The sensitivity and specificity of various combined TB-SS to predict TB were computed, and 95% confidence interval (CI) was included by using bootstrap method (resampling = 500) [26]. The positive likelihood ratio was also presented for each combination of TB-SS. TB-SS variables with high sensitivity were selected for further analysis. Choosing TB-SS with high sensitivity was to be comparable with social pathology characteristics for assessing which one had a better TB prediction on using as the TB screening tool.

To find out role of social pathology characteristics on TB prediction, this study was analyzed in accordance with guidelines for transparent reporting of a multivariable prediction model for individual prognosis or diagnosis (TRIPOD) statement for prediction studies [27].

#### Developing various TB screening models

The various TB screening models were developed to predict the bacteriologically confirmed TB: (i) combined models which include all social pathology characteristics and TB-SS variables with high sensitivity, (ii) social pathology model which include all social pathology characteristics variables, and (iii) TB-SS models which include TB-SS variables with high sensitivity.

#### Selecting candidate predictor variables for each TB screening model

For each TB screening model, association between predictor variables and bacteriologically confirmed TB was assessed by using chi-square test in a univariate analysis. Multivariate logistic regression analysis was performed to develop a predictive model of TB by including variables with the significant *p* value ≤ 0.2 in univariate analysis. The final multivariate model was chosen by stepwise backward method using Akaike information criterion (AIC). The model with the lowest AIC was the best for TB prediction. The significant level of variables to predict TB was set at *p* value < 0.05 [28].

The population attributable fraction (PAF) for each predictor variable was also calculated to assess the public health impact of social pathology characteristics in population for TB occurrence by using Miettinen’s formula [29] which is defined as follows:

$$ \mathrm{PAF}=\frac{p\times \left(\mathrm{ORadj}-1\right)}{\mathrm{ORadj}} $$

where *p* is the prevalence of TB for each predictor variable and ORadj is the adjusted odds ratio determined from the logistic regression model.

#### Calculating predicted probability of propensity score for TB risk in each TB screening model

In each TB screening tool, the regression coefficients of the significant variables in the final multivariate logistic regression model were used to drive a propensity score which is the conditional predicted probability of being diagnosed with a specific disease given values of covariates [30]. The propensity score summarizes all the relevant characteristics to predict disease in a single composite score [31]. Each participant was allocated with propensity score. The mathematical equation for calculating the propensity score from regression coefficients of the final logistic regression model was as follows [30]:

$$ \mathrm{Propensity}\ \mathrm{score}=\left(\exp \left(\upbeta 0+\upbeta 1\mathrm{X}1+\dots +\upbeta \mathrm{pXp}\right)\right)/\left(1+\exp \left(\upbeta 0+\upbeta 1\mathrm{X}1+\dots +\upbeta \mathrm{pXp}\right)\right) $$

#### Assessing the performance of each TB screening model to detect TB

To highlight the role of social pathology characteristics on TB screening, the performance of TB screening models were compared by measures of overall performance, calibration, and discrimination ability of propensity score to predict TB [32].

The overall performance of a TB screening tool was measured for the difference between the observed outcome and predicted probability of propensity score in TB by using the Nagelkerke R^2^ and Brier score [32]. Nagelkerke R^2^ explains variations of TB prediction by a model [33]. The Brier score ranges from 0 for a perfect model to 0.25 for a non-informative model to predict TB [34].

A model calibration for assessing the degree of consistency between observed outcome and predicted probability of propensity score in TB screening tool was performed based on the Hosmer–Lemeshow goodness-of-fit test with measurements of maximum absolute error (Emax) and mean absolute error (Eavg) as well as visually by plotting the observed TB cases against the predicted probability of propensity score in TB by 10% risk of stratification level [32, 35, 36]. A good calibration is when Hosmer–Lemeshow test yielded nonsignificant statistical value, as well as Emax and Eavg is zero, indicating no error or no difference between observed data and predicted propensity score value [37]. The calibration plot includes an intercept, which indicates the extent that predictions are systematically too low or too high “calibration-in-the-large,” and a calibration slope [36]. Having an intercept as zero and a slope as one indicates that the model is fit with perfect prediction of propensity score on the 45° line of plot [32].

Discrimination ability of propensity score between participants with and without TB in each TB screening model was assessed by using a receiver operator characteristic curve (ROC) analysis with area under the curve (AUC) or c-statistic [32]. The ROC curve shows the tradeoff between the sensitivity and the specificity of a classifier for various choices of the probability threshold of propensity score to binary outcome of bacteriologically confirmed TB. The AUC or c-statistics indicate the rank correlation between predicted probabilities of outcome occurring and the observed response. If ROC curve is close to upper left corner of the plot (sensitivity = 100 and specificity = 100%), then AUC or c-statistics closes to 100%. An AUC or c-statistic of < 70% represents poor discrimination while 80–90% indicates excellent discrimination [38]. The *p* value < 0.05 of DeLong’s test was used to show the significant difference in the AUC of models. In addition to AUC statistic, discrimination slope was calculated for how participants were separated with and without the TB by measuring difference in average predicted probability of propensity score to TB between them and also visually by box plot to show overlapping of predicted probability of propensity score between participants with and without TB [32, 39].

#### Model validation

Special focus was given to the influence of TB screening by social pathology characteristics. Therefore, internal validity of the final multivariate model with significant social pathology characteristic variables was assessed by bootstrap method (resampling = 500) [27]. To assess model fit and optimism, bootstrapped estimates of overall performance, calibration, and discrimination were compared with the original model’s estimates.

#### Sensitivity, specificity, and positive likelihood ratio of different propensity score cutoff values for various TB screening models by using bacteriologically confirmed TB as gold standard

The different cutoff values of propensity score in combined models and social pathology TB screening model were tabulated against bacteriologically confirmed TB as gold standard to calculate the sensitivity, specificity, and positive likelihood ratio which were compared with TB-SS models.

## Results

### Prevalence of bacteriologically confirmed TB summarized by TB signs and symptoms (TB-SS) and chest X-ray (CXR) as a parallel screening tool

Table 1 shows the ability of any TB-SS and CXR in detecting bacteriologically confirmed TB. Overall, 311 out of 51,367 participants had bacteriologically confirmed TB, equating to 605 cases per 100,000 populations. Almost all bacteriologically confirmed TB were initially detected in CXR screening, but 6 (1.9%) had a normal CXR. Of the 311 bacteriologically confirmed TB, 116 (37.3%) were asymptomatic.

**Table 1:** Distribution of bacteriologically confirmed TB detected using chest X-ray abnormality and any tuberculosis signs and symptoms as parallel screening tools

Screening tools		Total (*N* = 51,367)	Bacteriologically confirmed TB (*n*)		Bacteriologically confirmed TB (rate per 100,000 population)
			Positive	Negative	
			*N* = 311	*N* = 51,056	
Chest X-ray abnormality	Any TB signs and symptoms				
-	-	28,878	0	28,878^a^	0
-	+	11,852	6	11,846	50
+	-	5,555	116	5,439	2,088
+	+	5,082	180	4,902	3,541

^a^Those participants were categorized as normal healthy participants without proceeding any TB confirmation test as WHO guideline [23]

### Sensitivity, specificity, and positive likelihood ratio of various combinations of TB-SS by using bacteriologically confirmed TB as the gold standard

Table 2 shows the sensitivity, specificity, and positive likelihood ratio of various combinations of TB-SS by using bacteriologically confirmed TB as the gold standard. The table is sorted in descending order of frequency of occurrence. Out of 32 various combinations of TB-SS, having any signs and symptoms gave the most meaningful sensitivity of 59.8% (bootstrapped 95% CI 54.1–65.3) and specificity of 67.2% (bootstrapped 95% CI 66.7–67.2), but it was lower than CXR alone (sensitivity = 95.1% and specificity = 86.3%, shown in Table 1). The combination with the next best accuracy was any cough with a sensitivity of 24.4% (bootstrapped 95% CI 19.7–29.6) and specificity of 85% (bootstrapped 95% CI 85.5–86.1). The positive likelihood ratio > 1 result is the evidence to have positive bacteriologically confirmed TB if any TB signs and symptoms or cough is present. The remaining combinations of TB-SS had poor sensitivity (< 10%) compared to any TB-SS and any cough. The TB-SS having persistent cough > 2 weeks and other symptoms including night sweet also had the sensitivity of 8.4% and 5.8% and the specificity of 98.3% and 95.2%, respectively).

**Table 2:** Sensitivity, specificity, and positive likelihood ratio of various combinations of TB-SS by using bacteriologically confirmed TB as the gold standard

Category^a^	Cough	Hemoptysis	Recent weight loss	Chest pain	Fever	Total (*N*)	Bacteriologically confirmed TB						Positive likelihood ratio
							Positive (*N* = 311)			Negative (*N* = 51,056)			
							*n*	Sensitivity (%)	95% CI	*n*	Specificity (%)	95% CI	
1	Any TB signs and symptom					16,934	186	59.8	54.1–65.3	16,748	67.2	66.7–67.6	1.8
2	+	-	-	-	-	7306	76	24.4	19.7–29.6	7230	85.8	85.5–86.1	1.7
3	-	-	-	+	-	2861	8	2.6	1.1–5.0	2853	94.4	94.2–94.6	0.5
4	+	-	-	+	-	2312	26	8.4	5.5–12.0	2286	95.5	95.3–95.7	1.8
5	+	-	-	-	+	1005	12	3.9	2.0–6.6	993	98.1	97.9–98.2	1.9
6	-	-	-	-	+	819	9	2.9	1.3–5.4	810	98.4	98.3–98.5	1.7
7	+	-	-	+	+	598	11	3.5	1.7–6.2	587	98.9	98.7–98.9	1.8
8	-	-	+	-	-	410	8	2.6	1.1–5.0	402	99.2	99.1–99.3	3.2
9	+	-	+	+	-	401	8	2.6	1.1–5.0	393	99.5	99.4–99.6	3.3
10	-	-	-	+	+	299	1	0.3	0.0–1.7	298	99.4	99.3–99.5	0.5
11	-	-	+	+	-	168	0	0	0.0–1.1	168	99.6	99.5–99.7	0.0

“+” presence, “-” absence, *95% CI* 95% confidence interval (Bootstrap resampling = 500)

^a^The remaining 21 categories, which had a sensitivity of < 10% and specificity of ~ 99%, are not shown

### Selecting candidate predictor variables for various TB screening models

Table 3 shows univariate predictor variables associated with bacteriologically confirmed TB. Out of 18 predictor variables, 15 variables showed significant association with TB in univariate analysis and then included in the multivariate analysis of each TB screening model.

**Table 3:** Predictor variables associated with bacteriologically confirmed TB in univariate analysis

Predictor variables	Bacteriologically confirmed TB		Total	*P* value
	Negative	Positive		
Total	51,056	311	51,367	
**Social characteristics**				
Age group (years)				< 0.001
15–24	11,888 (23.3)	11 (3.5)	11,899 (23.2)	
25–34	11,172 (21.9)	52 (16.7)	11,224 (21.9)	
35–44	10,386 (20.3)	76 (24.4)	10,462 (20.4)	
45–54	8214 (16.1)	66 (21.2)	8280 (16.1)	
55+	9396 (18.4)	106 (34.1)	9502 (18.5)	
Gender				< 0.001
Female	28,868 (56.5)	105 (33.8)	28,973 (56.4)	
Male	22,188 (43.5)	206 (66.2)	22,394 (43.6)	
Education				< 0.001
Illiterate	5569 (10.9)	55 (17.7)	5624 (10.9)	
Read and write	5027 (9.8)	37 (11.9)	5064 (9.9)	
Primary	19,481 (38.2)	91 (29.3)	19,572 (38.1)	
Middle	11,184 (21.9)	66 (21.2)	11,250 (21.9)	
High	6549 (12.8)	44 (14.1)	6593 (12.8)	
University	1157 (2.3)	3 (1)	1160 (2.3)	
Graduate	2089 (4.1)	15 (4.8)	2104 (4.1)	
Occupation				< 0.001
Non farmer	17,677 (34.6)	105 (33.8)	17,782 (34.6)	
Farmer	22,784 (44.6)	113 (36.3)	22,897 (44.6)	
Dependent	10,595 (20.8)	93 (29.9)	10,688 (20.8)	
Religion				< 0.001
Buddhist	46,839 (91.7)	263 (84.6)	47,102 (91.7)	
Other religion	4217 (8.3)	48 (15.4)	4265 (8.3)	
Marital status				< 0.001
Single	14,914 (29.2)	39 (12.5)	14,953 (29.1)	
Married	31,669 (62)	226 (72.7)	31,895 (62.1)	
Separated/divorced	714 (1.4)	6 (1.9)	720 (1.4)	
Widow/widower	3759 (7.4)	40 (12.9)	3799 (7.4)	
Area of residence				< 0.001
Rural	39,905 (78.2)	208 (66.9)	40,113 (78.1)	
Urban	11,151 (21.8)	103 (33.1)	11,254 (21.9)	
Administrative division				< 0.001
Region	36,971 (72.4)	192 (61.7)	37,163 (72.3)	
State	14,085 (27.6)	119 (38.3)	14,204 (27.7)	
Smoking				< 0.001
Never smoked	33,412 (65.4)	135 (43.4)	33,547 (65.3)	
Smoked in the past	3016 (5.9)	49 (15.8)	3065 (6.0)	
Current smoker	14,628 (28.7)	127 (40.8)	14,755 (28.7)	
Drinking				< 0.001
Never drank	40,077 (78.5)	191 (61.4)	40,268 (78.4)	
Drank in the past	2957 (5.8)	49 (15.8)	3006 (5.9)	
Current drinker	8022 (15.7)	71 (22.8)	8093 (15.8)	
Contact with a known TB case				< 0.001
No	47,425 (93.3)	265 (85.8)	47,690 (93.3)	
Yes	3389 (6.7)	44 (14.2)	3433 (6.7)	
Previous history of TB				< 0.001
No	49,635 (97.2)	269 (86.5)	49,904 (97.2)	
Yes	1421 (2.8)	42 (13.5)	1463 (2.8)	
**Pathological characteristics**				
Body mass index (kg/m2)				< 0.001
Normal	32,821 (64.3)	149 (47.9)	32,970 (64.2)	
Underweight	11,689 (22.9)	147 (47.3)	11,836 (23.1)	
Overweight/obese	6511 (12.8)	15 (4.8)	6526 (12.7)	
History of diabetes mellitus				0.153
No	50,676 (99.3)	306 (98.4)	50,982 (99.3)	
Yes	380 (0.7)	5 (1.6)	385 (0.7)	
History of hypertension				0.404
No	45,646 (89.4)	273 (87.8)	45,919 (89.4)	
Yes	5410 (10.6)	38 (12.2)	5448 (10.6)	
History of HIV				1
No	51,047 (100)	311 (100)	51,358 (100)	
Yes	9 (0)	0 (0)	9 (0)	
**TB-SS**				
Any TB signs and symptoms			< 0.001	
Absence	34,308 (67.2)	125 (40.2)	34,433 (67)	
Presence	16,748 (32.8)	186 (59.8)	16,934 (33)	
Any cough				< 0.001
Absence	43,826 (85.8)	235 (75.6)	44,061 (85.8)	
Presence	7230 (14.2)	76 (24.4)	7306 (14.2)	

*HIV* human immunodeficiency virus, *TB* tuberculosis, *TB-SS* tuberculosis signs and symptoms

Table 4 reveals that the five TB screening models, A to E, were developed to predict TB by logistic regression analysis. Models A and B were the combined models including the significant social pathology characteristics and TB-SS variables (any TB signs and symptoms and any cough, respectively), and model C was our proposed social pathology model including significant social pathology characteristic variables while model D and E were TB-SS models including any TB signs and symptoms variable and any cough variable, respectively.

**Table 4:** Various TB screening models to predict bacteriologically confirmed TB in logistic regression analysis

Predictor variables	Various TB screening models to predict bacteriologically confirmed TB				
	Combined models		Social pathology model	TB-SS models	
	A	B	C	D	E
**I. Predictor variables to predict bacteriologically confirmed TB in univariate analysis**					
(1) All social pathology characteristics variables	+	+	+		
(2) Any TB signs and symptoms variable	+			+	
(3) Any cough variable		+			+
**II. Coefficient (β) of significant predictor variables to predict bacteriologically confirmed TB in final multivariate logistic regression analysis**					
Intercepts	− 8.7262	− 8.5302	− 8.4746	− 5.6148	− 5.22840
**Social characteristics**					
Age group (years)				-	-
15–24	0.0000	0.0000	0.0000		
25–34	1.7756	1.8222	1.8248		
35–44	2.3192	2.3699	2.3756		
45–54	2.3008	2.3831	2.3916		
55+	2.2943	2.3852	2.4088		
Gender				-	-
Female	0.0000	0.0000	0.0000		
Male	1.0400	1.0261	1.0436		
Occupation				-	-
Non farmer	0.0000	0.0000	0.0000		
Farmer	− 0.2440	− 0.2198	− 0.2130		
Dependent	0.4153	0.4241	0.4323		
Religion				-	-
Buddhist	0.0000	0.0000	0.0000		
Other	0.4001	0.4663	0.4893		
Area of residence				-	-
Rural	0.0000	0.0000	0.0000		
Urban	0.5039	0.4673	0.4520		
Administrative division				-	-
Region	0.0000	0.0000	0.0000		
State	0.3727	0.4529	0.4664		
Contact with a known TB case				-	-
No	0.0000	0.0000	0.0000		
Yes	0.6562	0.7116	0.7214		
Previous history of TB				-	-
No	0.0000	0.0000	0.0000		
Yes	0.9422	1.0226	1.0313		
**Pathology characteristic**					
Body mass index				-	-
Underweight	0.9923	1.0379	1.0519		
Normal	0.0000	0.0000	0.0000		
Overweight/obese	− 0.8556	− 0.9038	− 0.9069		
**TB-SS**					
Any TB signs and symptoms					
Absence	0.0000			0.0000	
Presence	0.8601			1.1145	
Any cough					
Absence		0.0000			0.0000
Presence		0.4682			0.67314
AIC	3331.4	3373	3382.1	3706.3	3777.8
Median (IQR) of propensity score	0.003 (0.001–0.006)	0.003 (0.001–0.007)	0.003 (0.001–0.006)	0.003 (0.002–0.01)	0.005 (0.004–0.006)

All social pathology variables included age, gender, education, occupation, religion, marital status, area of residence (rural or urban), administrative division (region or state), smoking and drinking, contact with a known TB case, previous history of TB, body mass index (kg/m^2^), diabetes mellitus, human immunodeficiency virus and hypertension. Variables included in univariate analysis of each TB screening model are indicated by “+”. The propensity score for each model was calculated from coefficient (β) of significant predictor variables in each TB screening model

*AIC* Akaike information criterion, *IQR* interquartile range, *TB* tuberculosis, *TB-SS* TB signs and symptoms

In the final multivariate analysis, the combined models (A and B) were the best fit with AIC = 3331 and AIC = 3373, respectively while social pathology model (C) was the best fit with AIC = 3382 for TB prediction. The significant social pathology characteristic variables associated with TB in models A, B, and C were the same such as higher age group (years), being male, dependent, other religion, living in urban area of residence, living in state administrative division, having contact with a known case of TB, having previous history of TB, and underweight. Table 5 shows that the three factors having the highest population attributable fraction to predict TB were male gender (42.6%), age ≥ 55 years (31.0%), and underweight (30.4%).

**Table 5:** Social pathology predictor variables associated with bacteriologically confirmed TB and their population attributable fraction

Covariates	Bacteriologically confirmed TB (*N* = 311)	Crude odds ratio (95% CI)	Adjusted odds ratio (95% CI)	*p* value	Population attributable fraction (%)
Age group (years)				< 0.001	
15–24	11	Ref	Ref		-
25–34	52	4.9 (2.5, 9.4)	6.2 (3.2, 11.9)		14.0
35–44	76	7.9 (4.2, 14.8)	10.7 (5.6, 20.3)		22.2
45–54	66	8.5 (4.5, 16.2)	10.9 (5.7, 20.8)		19.3
55+	106	12.2 (6.5, 22.7)	11.1 (5.9, 20.9)		31.0
Gender				< 0.001	
Female	105	Ref	Ref		-
Male	206	2.5 (2.0, 3.2)	2.8 (2.2, 3.6)		42.6
Occupation				< 0.001	
Non farmer	105	Ref	Ref		
Farmer	113	0.8 (0.6, 1.1)	0.8 (0.6, 1.0)		-
Dependent	93	1.4 (1.1, 1.9)	1.5 (1.1, 2.1)		10.0
Religion				0.007	
Buddhist	263	Ref	Ref		-
Other	48	2.0 (1.5, 2.8)	1.6 (1.1, 2.3)		5.8
Rural and urban residences				0.004	
Rural	208	Ref	Ref		-
Urban	103	1.7 (1.3, 2.2)	1.5 (1.2, 2.0)		11.0
Regions and states				< 0.001	
Region	192	Ref	Ref		-
State	119	1.6 (1.2, 2.0)	1.6 (1.2, 2.0)		14.3
Contact with TB				< 0.001	
No	265	Ref	Ref		-
Yes	44	2.3 (1.6,3.2)	2.0 (1.4,2.8)		7.1
Previous history of TB					
No	269	Ref	Ref		-
Yes	42	5.5 (3.9, 7.7)	2.8 (1.9, 3.9)	< 0.001	8.7
Body mass index group				< 0.001	
Underweight	147	2.7 (2.2, 3.5)	2.8 (2.2, 3.6)		30.4
Normal	149	Ref	Ref		-
Overweight/obese	15	0.4 (0.2, 0.8)	0.4 (0.2, 0.6)		− 6.0

*TB* tuberculosis, *CI* confidence interval

### Calculating predicted probability of propensity score for TB risk in each TB screening model

Table 4 shows that the predicted probability of propensity score for bacteriologically confirmed TB for each TB screening model was derived from coefficients of significant variables in final multivariate regression analysis.

### Assessing the performance of each TB screening model to detect TB by using predicted propensity score

**Fig. 2:**
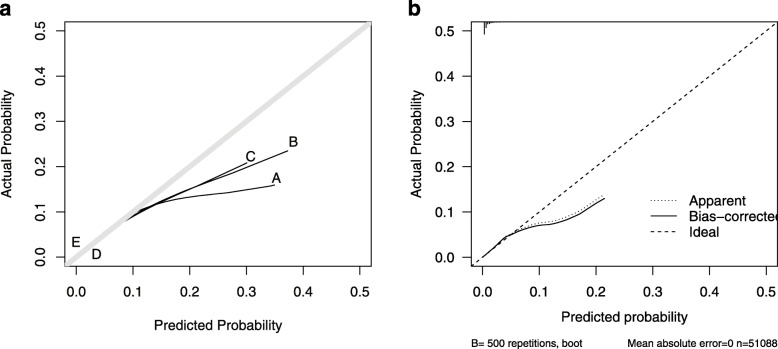
**a** Calibration plot of various TB screening models A to E. **b** Calibration plot of validated data of social pathology model C (Bootstrap resampling=500)

**Fig. 3:**
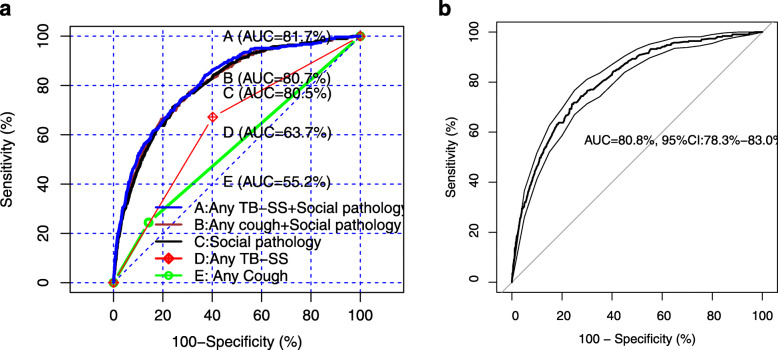
**a** ROC curve of various TB screening models A to E. **b** ROC curve of validated data of social pathology model C (Bootstrap resampling=500)

**Fig. 4:**
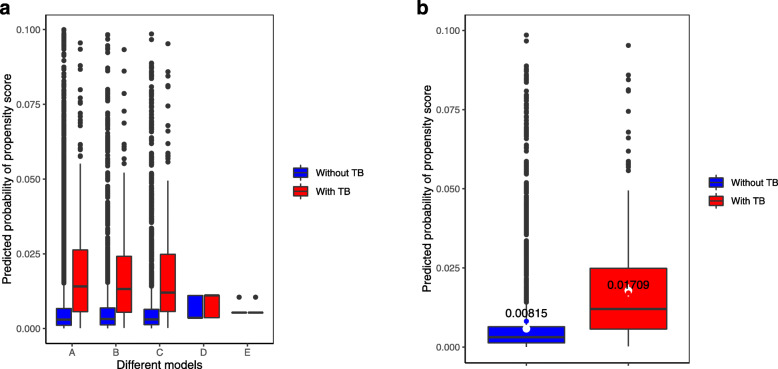
**a** Discrimination box plot of various TB screening models A to E. **b** Discrimination box plot of social pathology model C (Bootstrap resampling=500)

Table 6 shows overall performance, calibration, and discrimination ability of predicted propensity score in various TB screening models to predict bacteriologically confirmed TB. Figures 2a, 3a, and 4a show virtual plot for calibration and discrimination slope of box plot for TB screening models A to E while Figs. 2b, 3b, and 4b were plots for internal validation of the proposed social pathology model C (bootstrap resampling = 500).

**Table 6:** Overall performance, calibration, and discrimination ability of predicted propensity score in various TB screening models to predict bacteriologically confirmed TB

Model performance measures	Various TB screening models to predict bacteriologically confirmed TB					
	Combined models		Social pathology model	TB-SS models		Validated data of social pathology model (Bootstrap resampling = 500)
	A	B	C	D	E	C
**Overall performance**						
Nagelkerke R^2^	12.9%	11.8%	11.5%	2.7%	0.6%	11.9% (95% CI 9.8–13.8%)
Brier score	0.0059	0.0059	0.006	0.006	0.006	0.0058 (95% CI 0.005–0.006)
**Calibration**						
Hosmer–Lemeshow test	0.005	0.604	0.787	1	1	0.502
Slope	1	1	1	1	1	1
Intercept	0	0	0	0	0	0
Eavg	0.001	0.000	0.000	0.000	0.000	0.0006 (95% CI 0.0003–0.001)
Emax	0.190	0.138	0.093	0.000	0.000	0.126 (95% CI 0.03–0.295)
**Discrimination**						
AUC	81.7	80.7	80.5	63.7	55.2	80.8 (95% CI 78.3–83.0)
DeLong *p* value	0.0136	0.3606	Reference	0.0001	0.0001	
Discrimination slope	0.018	0.015	0.015	0.002	0.001	0.012 (95% CI 0.004–0.018)

Combined model A includes significant social pathology characteristics and any TB-SS variable. Combined model B includes significant social pathology characteristics and any cough variable. Model C includes significant social pathology characteristics. TB-SS model D includes any TB-SS variable. TB-SS model E include any cough variable

*AUC* area under the curve, *Eavg* average absolute error, *Emax* maximal absolute error, *95% CI* 95% confidence interval by bootstrap resampling = 500

The overall performance R^2^ was high in combined models (A, 12.9% and B, 11.8%) and social pathology model (C, 11.5%) while it was low in TB-SS models (D, 2.7% and E, 0.6%). Brier score was close to zero, indicating all models were perfect and informative to predict bacteriologically confirmed TB.

Figure 2a shows that a better calibration, more closer to 45° line of plot, was observed in the social pathology model (C) and TB-SS models (D and E), compared to combined models (A and B). In the original and validated bootstrapping social pathology models (C), the Hosmer-Lemeshow test yielded nonsignificant statistics (*p* value = 0.787 and 0.502, respectively) with zero in Eavg, Emax, and intercept and one in slope, suggesting that there was no departure from perfect fit between prediction and observed value.

Figure 3a reveals that the discrimination ability was excellent in combined models (A, AUC = 81.7% and B, AUC = 80.7%) and social pathology model (C, AUC = 80.5% with bootstrapped 95% CI 78.3–83.0%) while it was poor in TB-SS models (D, AUC = 63.7% and E, AUC = 55.2%). The social pathology model showed little evidence of overfitting that is optimism in estimated AUC between original and validated bootstrapping model was 0.003. The social pathology model significantly improved the discrimination ability, compared to TB-SS models (DeLong *p* value = 0.0001).

In Fig. 4a, the discrimination slope in box plot shows that overlapping the predicted probability of propensity score between participants with and without TB was less likely to be in combined models (A and B) and social pathology model (C) while it was more likely to be in TB-SS models (D and E). The discrimination slope of the social pathology model (C) was 0.015 with bootstrapped 95% CI 0.004 to 0.018.

### Sensitivity, specificity, and positive likelihood ratio of different propensity score cutoff values in various TB screening models by using bacteriologically confirmed TB as the gold standard

Table 7 shows the propensity score of 0.0053 and above cutoff level in combined models (A and B), and social model (C) had higher sensitivity, ~ 80% to predict TB compared to TB-SS models (D, 59.8% and E, 24.4%) while those had the specificity, ~ 70% higher than the TB-SS model with any TB-SS variable (D, 67%). The propensity score cutoff level used to define high-risk increases, the sensitivity decreases but the positive likelihood ratio increases, indicating that the screening test of the proposed social pathology model could be used to clearly rule-in or rule-out the risk of TB.

**Table 7:** Sensitivity, specificity, and positive likelihood ration to predict TB by different cutoff points of propensity score in various TB screening models

Cutoff points of propensity score (< vs ≥)	Total (*N*)	Bacteriologically confirmed TB						Positive likelihood ratio
		Positive (*N* = 311)			Negative (*N* = 51,056)			
		*n*	Sensitivity (%)	95% CI	*n*	Specificity (%)	95% CI	
Combined model A								
0.001	39,230	299	97.0	94.0–98.0	38,931	23.0	23.0–24.0	1.3
0.005	16,314	239	77.0	72.0–82.0	16,075	68.0	68.0–69.0	2.4
0.0053	15,567	235	76.0	71.0–81.0	15,332	70.0	69.0–70.0	2.5
0.01	7926	185	60.0	54.0–65.0	7741	85.0	84.0–85.0	3.9
0.05	487	38	12.0	9.0–16.0	449	99.0	98.9–99.9	13
Combined model B								
0.001	39,688	300	97.0	95.0–99.0	39388	22.0	22.0–23.0	1.3
0.005	17,504	245	79.0	74.0–84.0	17259	66.0	65.9–66.9	2.3
0.0053	16,132	239	77.0	72.0–82.0	15893	69.0	68.0–69.9	2.5
0.01	7547	176	57.0	51.0–63.0	7371	85.0	85.0–86.0	3.9
0.05	441	27	9.0	6.0–12.0	414	99.0	98.9–99.9	10.7
Model C								
0.001	40,546	301	97.0	95.0–99.0	40,245	21.0	20.0–21.0	1.2
0.005	20,478	256	83.0	78.0–87.0	20,222	60.0	60.0–61.0	2.1
0.0053	16,738	238	77.0	72.0–82.0	16,500	68.0	67.0–68.9	2.4
0.01	8936	190	61.0	56.0–67.0	8746	83.0	82.0–83.0	3.6
0.05	360	22	7.0	5.0–11.0	338	99.0	89.9–99.9	10.7

Combined model A includes significant social pathology characteristics and any TB-SS variable. Combined model B includes significant social pathology characteristics and any cough variable. Model C includes significant social pathology characteristics

## Discussion

Almost all bacteriologically confirmed TB were initially detected in CXR screening but only 2% of confirmed cases were missed. Over one third of TB cases were asymptomatic. The conventional TB-SS screening tool using any TB signs and symptoms had low sensitivity and specificity, compared to using CXR and social pathology characteristic screening tools. Use of social pathology characteristics regardless of TB-SS in a predictive model had good calibration and could outperform in discrimination ability to predict TB compared to any TB signs and symptoms or any cough.

In our study, nearly all bacteriologically confirmed TB cases in this study were initially suspected for abnormal CXR findings; less than 2% had a normal CXR—a finding consistent with other studies [40, 41]. WHO recommends the use of initial CXR followed by an acid-fast bacilli smear and Gene Xpert test if the CXR is abnormal [42]. In Myanmar, CXR and diagnostic radiologists are only available at township level hospitals covering an average population of around 150,000–200,000 [43]. With a TB prevalence of 242 per 100,000 population in 2018, annual CXR may be justifiable [44]. Given CXR is still not adequately available due to limitations of human and material resources as in other countries, a screening tool is needed for referring presumptive TB with symptoms to the health system [45].

Therefore, we analyzed the use of single or multiple TB symptoms as the rule-in criteria for the first TB screening step. Our findings revealed that over one third of TB cases had no TB symptoms, and conventional TB-SS screening via any TB signs and symptoms had low sensitivity and specificity compared to CXR and social pathology characteristics screening tools. Having low sensitivity was because people were more likely to have low tolerance to tuberculosis signs and symptoms in response to TB infection [46, 47]. On the other hand, having low specificity was more likely because the majority of those with tuberculosis signs and symptoms may have had other underlying conditions such as acute viral bronchitis, chronic bronchitis, and respiratory complications that lead to an overuse of diagnostic tests among individuals without TB [48–50].

Finally, in addition to TB-SS screening tools, development of new screening approach with prediction models have been increasingly used to improve TB case detection but did not perform any comparison to highlight the role of social pathological characteristics in TB screening [28, 51, 52]. We developed various TB screening models by using logistic regression analysis, and their performance on TB prediction was compared. Our proposed model of social pathology characteristics shows better calibration than combined models of social pathology characteristics and TB-SS and shows higher discrimination ability than TB-SS models. Many studies revealed that a useful screening tool is considered to have applied only when calibration and discrimination are good in performance [32, 37, 53].

All significant social pathology characteristic variables in the proposed social pathology model were consistent with the findings from the previous National TB prevalence survey in different countries [54–57]. However, our proposed social pathology model requires external validation in order to confirm that it predicts well in general population outside of our dataset. Assuming external validity, the decision-makers have to consider local needs by weighting sensitivity, specificity, and TB prevalence to choose appropriate cutoff value for TB prediction [58, 59]. Our study shows that using propensity score cutoff value ≥ 0.0053, which was driven from the significant social pathology characteristics, had a sensitivity of about 80% and specificity of nearly 70% to detect bacteriologically confirmed TB. Therefore, a particle way to use the significant social pathology characteristics as a TB screening tool was to choose the appropriate propensity score cutoff value for TB prediction. After that, chest X-ray and sputum examinations should be performed for TB confirmation. Using the scoring system in the TB prediction has been documented in some studies [10, 60, 61].

Our study has several strengths in consideration of improving TB screening in the general population. Firstly, the predicted social pathology characteristics in our proposed TB screening model are easily measurable when conducting the community-based TB screening program. Secondly, the significant social pathology characteristics have strong evidence on association with TB in many diverse locations, indicating the important role of the social pathology characteristics on TB screening tool across the wide range of settings [54–57]. Thirdly, our study used large sample size of national TB prevalence survey data to develop and validate model in accordance with TRIPOD guideline [27].

Our study has some limitations. Firstly, all participants with both normal chest X-ray and without any TB signs and symptoms were categorized as the healthy participants without preceding any confirmation test for TB. The negative result in the combined test of CXR and symptom screening does not need to conduct further testing, as TB prevalence in this group is very low, that is less likely to distort the sensitivity and specificity of our findings [23]. Secondly, we could not consider cost effectiveness of TB screening models that are also important for programmatic implementation.

Using the significant social pathology characteristics as the TB screening tool had good calibration and had improved the discrimination ability to 80.5%, when comparing to 63% in using TB-SS, indicating the important role of the significant social pathology characteristics on TB screening. Our study highlights the need for improving the existing TB screening tool endorsed by the National Tuberculosis Programme, especially for the areas with the high prevalence of TB.

## Conclusions

Incorporating the significant social pathology characteristics substantially improved the accuracy of TB screening. The National TB screening standard practice should therefore be changed accordingly.

## Data Availability

The datasets used and/or analyzed during the current study are available from the National TB program, Myanmar, on reasonable request.

## Abbreviations

AIC: Akaike information criterion
AUC: Area under the curve
95% CI: 95% confidence interval
CXR: Chest x-ray
HIV: Human immunodeficiency virus
IQR: Interquartile range
ROC: Receiver operating characteristics
TB: Tuberculosis
TB-SS: Tuberculosis signs and symptoms
